# Withanolide D Exhibits Similar Cytostatic Effect in Drug-Resistant and Drug-Sensitive Multiple Myeloma Cells

**DOI:** 10.3389/fphar.2017.00610

**Published:** 2017-09-08

**Authors:** Mark E. Issa, E. M. K. Wijeratne, A. A. L. Gunatilaka, Muriel Cuendet

**Affiliations:** ^1^School of Pharmaceutical Sciences, University of Geneva, University of Lausanne Geneva, Switzerland; ^2^Natural Products Center, School of Natural Resources and the Environment, College of Agriculture and Life Sciences, The University of Arizona, Tucson AZ, United States

**Keywords:** withanolide D, multiple myeloma cancer stem cells, resistance, efflux transporters, P-glycoprotein

## Abstract

In spite of recent therapeutic advances, multiple myeloma (MM) remains a malignancy with very low curability. This has been partly attributed to the existence of a drug-resistant subpopulation known as cancer stem cells (CSCs). MM-CSCs are equipped with the necessary tools that render them highly resistant to virtually all conventional therapies. In this study, the growth inhibitory effects of withanolide D (WND), a steroidal lactone isolated from *Withania somnifera*, on drug-sensitive tumoral plasma cells and drug-resistant MM cells have been investigated. In MTT/XTT assays, WND exhibited similar cytostatic effects between drug-resistant and drug-sensitive cell lines in the nM range. WND also induced cell death and apoptosis in MM-CSCs and RPMI 8226 cells, as examined by the calcein/ethidium homodimer and annexin V/propidium iodide stainings, respectively. To determine whether P-glycoprotein (P-gp) efflux affected the cytostatic activity of WND, P-gp was inhibited with verapamil and results indicated that the WND cytostatic effect in MM-CSCs was independent of P-gp efflux. Furthermore, WND did not increase the accumulation of the fluorescent P-gp substrate rhodamine 123 in MM-CSCs, suggesting that WND may not inhibit P-gp at the tested relevant doses. Therefore, the WND-induced cytostatic effect may be independent of P-gp efflux. These findings warrant further investigation of WND in MM-CSC animal models.

## Introduction

Multiple myeloma is a PC neoplasm that accounts for 13% of hematological malignancies, and 1% of all cancers (Franqui-Machin et al., 2015). MM is a complex process by which asymptomatic monoclonal gammopathy of undetermined significance is transformed into a smoldering myeloma, which in turn progresses into symptomatic myeloma, and inevitably evolves into drug-resistant refractory myeloma, where therapeutic options become very limited (Franqui-Machin et al., 2015). This drug-resistant phenotype is attributed to a rare subpopulation of MM cells termed CSCs. MM-CSCs are believed to be the cause behind the high mortality rate observed among MM patients (Basak and Carrier, 2010; Hajek et al., 2013; Kellner et al., 2013; Franqui-Machin et al., 2015). CSCs can be identified based on surface stem cell markers such as CD44, CD133 or CD166, as well as on functional assays that measure the ability of CSCs to detoxify their intracellular environment. This ability is attributed to the high expression of metabolic enzymes such as aldehyde dehydrogenase and to ABC efflux transporters, a characteristic that renders MM-CSCs highly resistant to virtually all conventional MM therapies (Dean et al., 2005; Januchowski et al., 2013).

P-glycoprotein is highly expressed in the intestine, liver, kidneys, and blood-brain-barrier, a characteristic that highlights its important role in the protection of the human body from harmful substances (Dean et al., 2005). In addition, P-gp has been discovered to be expressed not only in CSCs but also in normal hematopoietic stem cells (NSCs) (Dean et al., 2005). P-gp-deficient mice do have a NSC compartment, but are more susceptible to the effects of chemotherapy (Dean et al., 2005). Interestingly, patients that have been previously treated with chemotherapy exhibit higher P-gp expression (Abraham et al., 2015). Although several generations of P-gp inhibitors have been developed, these have failed clinically due to severe adverse effects (Abraham et al., 2015). One novel strategy to circumvent the effects of P-gp in conferring resistance to MM-CSCs could be to identify novel anticancer therapeutics that exert their effects without dependence on P-gp efflux function. Such novel anticancer therapeutics may significantly improve the prognosis of MM patients, particularly relapsed ones.

The natural products reservoir continues to be a major source of novel chemical scaffolds leading to the identification of promising anticancer therapeutics (Mondal et al., 2012a). Withanolides constitute one such class of molecules. They are found in a number of species of Solanaceae including *Withania somnifera* (L.) Dunal, a plant commonly used in Ayurvedic traditional medicine (Mishra et al., 2000). *W. somnifera* is traditionally prescribed for inflammatory conditions such as arthritis and rheumatism, and as a herbal recipe for general health, sexual health, longevity, and geriatrics (Mishra et al., 2000; Mirjalili et al., 2009). It is also traditionally used to enhance strength, endurance and to increase the generation of blood and muscles (Mirjalili et al., 2009). Recent scientific reports claimed that *W. somnifera* ethanolic root extracts prevented skin carcinoma in Swiss albino mice (Prakash et al., 2002; Padmavathi et al., 2005).

**FIGURE 1:**
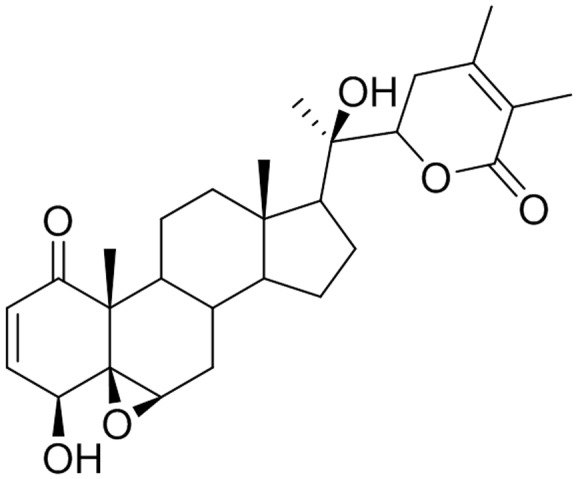
Chemical structure of WND.

Withanolide D is a steroidal lactone isolated from *W. somnifera* (**Figure 1**). WND has been reported to exhibit strong antileukemic effects through the induction of cell death and apoptosis in both dose-dependent and time-dependent manners in myeloid leukemia K562 cells, in the lymphoid leukemia MOLZ-4 cells, and in primary cells derived from leukemia patients (Mondal et al., 2010). In addition, WND inhibited tumor growth in K562 xenograft nude mice, and resulted in significant reduction of both tumor size and volume. Importantly, WND did not appear to cause any adverse effects (Mondal et al., 2012b).

The goal of the present study was to investigate the cytostatic effects of WND in drug-sensitive and drug-resistant myeloma cells. The influence of P-gp function on WND cytostatic activity was also addressed in MM-CSCs.

## Materials and Methods

### Chemicals and Biologicals

Withaferin A was purchased from Enzo Life Sciences (Lausen, Switzerland). Bortezomib, MTT, XTT, calcein, EH, VRP, and R123 were purchased from Sigma–Aldrich (Buchs, Switzerland).

### WND Isolation from *W. somnifera*

Dried and powdered roots of *W. somnifera* (1.6 kg) were extracted three times with CH_2_Cl_2_/MeOH (1:1, 4 L) (Xu et al., 2009). Extracts were combined and evaporated under reduced pressure to afford the crude extract (108.5 g) which was partitioned between 80% aq. MeOH and hexanes. The 80% aq. MeOH fraction which contained withanolides was diluted to 50% aq. MeOH with water and extracted with CHCl_3_. Evaporation of CHCl_3_ under reduced pressure gave an extract (22.1 g) enriched with withanolides. A portion (6.0 g) of this extract was subjected to size-exclusion chromatography over a column of Sephadex LH-20 (160.0 g) made up in hexanes/CH_2_Cl_2_ (1:4) and eluted with hexanes/CH_2_Cl_2_ (1:4, 1.5 L), CH_2_Cl_2_/acetone (3:2, 1.0 L), CH_2_Cl_2_/MeOH (1:1, 1.0 L), and finally with MeOH (0.5 L). Thirty fractions (125 mL each) were collected and combined based on their TLC profiles to yield 18 combined fractions [A (1133.8 mg), B (859.3 mg), C (334.6 mg), D (212.5 mg), E (134.4 mg), F (140.9 mg), G (182.4 mg), H (428.4 mg), I (280.9 mg), J (154.7 mg), K (43.1 mg), L (16.7 mg), M (65.8 mg), N (120.5 mg), O (350.8 mg), P (246.2 mg), Q (512.4 mg), and R (620.3 mg)]. Fraction G which contained the majority of withanolides was subjected to column chromatography over silica gel (3.5 g) made up in Et_2_O and eluted with Et_2_O followed by increasing amounts of MeOH in Et_2_O to give seven sub-fractions [G1 (3.4 mg), G2 (3.0 mg), G3 (1.5 mg), G4 (26.1 mg), G5 (82.5 mg), G6 (48.5 mg), and G7 (10.1 mg)]. The sub-fraction G4 on trituration with Et_2_O afforded WND as a white solid (18.2 mg); ${\left[\alpha \right]}_{D}^{25}$ +78 (*c* 0.2, MeOH) [liter +80] (Lavie et al., 1968). The ^1^H NMR, ^13^C NMR, and mass spectroscopic data were consistent with those reported (Roumy et al., 2010).

### Cell Culture

Multiple myeloma cancer stem cells were purchased from Celprogen (Torrance, CA, United States). They were isolated from a bone marrow lesion of a MM patient, and are CD44^+^, CD166^+^ and CD138^-^ (Medema, 2013; Issa et al., 2016; Issa and Cuendet, 2017). The tumorigenicity of MM-CSCs, as determined by Celprogen, is <1000 cells (Medema, 2013; Issa et al., 2016). MM-CSCs were maintained in the hematopoietic stem cell medium StemPRO-34 (Life Technologies, Zug, Switzerland), supplemented with 10% FBS (Nuaillé, France), 100 IU/mL penicillin and 250 μg/mL streptomycin (Life Technologies), and 2 mM L-glutamine. MM-CSCs were utilized at passages between 4 and 12 to preserve a stem cell state. The tumoral PCs RPMI 8226 (RPMIs), the DEX-sensitive MM1.S and the DEX-resistant MM1.R were obtained from LGC standards (Middlesex, United Kingdom), and were maintained in RPMI-1640 medium supplemented with 10% FBS, 100 IU/mL penicillin, and 250 μg/mL streptomycin. RPMIs are mainly composed of CD138^+^ cells, but have a fraction of 2–5% CD138^-^ cells known to be tumorigenic (Matsui et al., 2004). Normal hematopoietic CD34^+^ stem cells (NSCs) were purchased from Life Technologies, and maintained in StemPRO-34 medium, supplemented with Cytokine Mix E (PromoCell, Heidelberg, Germany) and 2 mM L-glutamine. NSCs were utilized at passages between 3 and 6 to preserve a stem cell state. MM-CSCs, NSCs, RPMIs, MM1.S, and MM1.R cells were passaged every 2–3 days, and were maintained in a humidified atmosphere supplemented with 5% CO_2_ at 37°C (standard conditions).

### MTT/XTT Assay

Multiple myeloma cancer stem cells were plated in 96-well plates at a density of 5,000 cells per well and allowed to adhere for 24 h, whereas RPMIs, MM1.S, and MM1.R cells were plated at a density of 15,000 cells per well and treated immediately with WND or positive controls. After 72 h incubation, cells were incubated for 2 h with 20 μL of MTT solution (5 mg/mL) for MM-CSCs, or for 4 h with 50 μL XTT solution (1 mg/mL) for RPMIs, MM1.S, and MM1.R cells. Cell growth was computed as the absorbance of each test well divided by that of the vehicle control wells and then multiplied by 100. The concentration that resulted in 50% reduction in cell growth (IC_50_) was computed from at least five different concentrations using GraphPad Prism 5.0 software.

### Viability/Mortality and Apoptosis Staining

Multiple myeloma cancer stem cells were plated in 6-well plates at a density of 100,000 cells per well and allowed to adhere for 24 h before WND treatment at which about 300,000 cells were present in the well. RPMIs and NSCs were seeded at 300,000 cells per well in 6-well plates and were treated immediately with increasing concentrations of WND for 24 h. Flow cytometric analyses were performed on MM-CSCs, RPMIs, and NSCs to examine the effects of WND on cell viability/mortality using calcein staining for viability and EH staining for mortality. For apoptosis, MM-CSCs and RPMIs were assessed after 24 h WND treatment using the AV/PI staining (Life Technologies). Following treatment with WND, floating and adherent cells were collected, washed with PBS, adjusted to 10^6^ cells/mL, and then incubated with the corresponding stain at room temperature for 15 min. Cells were then analyzed using the Attune NxT Acoustic Focusing Flow Cytometer (Applied Biosystems, Life Technologies, Zug, Switzerland).

### Rhodamine 123 Accumulation Assay

To determine whether or not WND was an inhibitor of P-gp efflux transport, R123 accumulation assays were performed. MM-CSCs were plated at a density of 15,000 cells per well in 96-well plates, and were allowed to adhere overnight. MM-CSCs were washed with PBS and incubated in 250 μL of 10 μM R123 solution with or without WND or controls for 30 min at 37°C. MM-CSCs were then washed once with PBS and incubated with 50 μL of 1 μL/mg DAPI stain. VRP was used as positive control inhibitor of P-gp efflux transport. MM-CSCs were then washed once with PBS and intracellular R123 fluorescence was immediately quantified using the Cytation 3 Cell Imaging Reader (Biotek, Winooski, VT, United States). The R123 fluorescence was normalized to DAPI nuclear stain. The R123 levels in drug-treated MM-CSCs were compared to that in vehicle control. The average normalized R123 accumulation was multiplied by 100 and divided by the average R123 accumulation vehicle control-treated MM-CSCs.

### Quantitative Real-time PCR Analysis of mRNA Expression

Multiple myeloma cancer stem cells, NSCs, and RPMIs were lysed, and total RNA was collected from cell lysates with or without WND treatment using the Aurum total RNA Mini Kit (Bio-Rad, Cressier, Switzerland). Reverse transcription was conducted using 0.5 μg RNA, the “high capacity total RNA to cDNA” Reverse Transcriptase (Life Technologies) and a PCR standard thermal cycler (Applied Biosystems). One microliter cDNA was amplified by quantitative PCR using a SYBR Green PCR Kit (Applied Biosystems) and a Step One Plus Real-Time PCR Thermal Cycler (Applied Biosystems). Custom primers for *GAPDH*, *18S*, and *P-gp* were designed on http://bioinfo.ut.ee/primer3-0.4.0/ and obtained from Life Technologies (Koressaar and Remm, 2007; Untergasser et al., 2012). Relative gene expression was calculated by normalizing to the average of two housekeeping genes *18S* and *GAPDH* using the ΔΔ*C*_T_ method.

### Statistical Analysis

Each experiment was conducted at least three times, and all data were expressed as mean ± SEM. An unpaired *t-*test was employed for statistical comparisons that involved two groups. A one-way ANOVA was employed for multiple comparisons in experiments with one independent variable, and a Bonferroni test was used for *post hoc* analysis. A significant difference in mean values between groups was considered when *p* < 0.05.

## Results

### WND Similarly Inhibited the Growth of Drug-Sensitive and Drug-Resistant MM Cells

Using MTT and XTT assays, the effect of WND on cell growth in MM-CSCs, RPMIs, MM1.S, and MM1.R cells was investigated. Results indicated that a 72 h WND treatment inhibited MM-CSCs growth with an IC_50_ value of 88.4 ± 13.9 nM, and that of RPMIs with an IC_50_ of 76.1 ± 8.0 nM (**Table 1**). The IC_50_ values of WND in MM1.S and MM1.R cells were 107.2 ± 14.2 nM and 106.3 ± 11.0 nM, respectively (**Table 1**). Statistical analysis indicated that the IC_50_ values of WND were similar between MM-CSCs and RPMIs, and between MM1.S and MM1.R cells. Results also showed that the IC_50_ values of WFA were significantly higher in the resistant cells, MM-CSCs and MM1.R, than in the sensitive ones RPMIs and MM1.S (**Table 1**) (Issa and Cuendet, 2017). The behavior of BTZ, a control drug, was similar to that of WFA. This shows the resistant phenotype in MM-CSCs and MM1.R cells (**Table 1**).

**Table 1:** Antiproliferative activity (IC_50_ in nM) of WND, WFA, and BTZ in MM cells.^a^

Compound	MM-CSCs	RPMIs	MM1.R	MM1.S
WND	88.4 ± 13.9	76.1 ± 8.0	106.3 ± 11.0	107.2 ± 14.2
WFA^b^	547.3 ± 64.9^∗^	257.7 ± 23.8	262.7 ± 20.4^∗^	52.8 ± 9.7
BTZ^b^	8.1 ± 0.8^∗^	4.7 ± 0.3	6.7 ± 0.7^∗^	4.7 ± 0.3


*^a^The antiproliferative activity of WND, WFA, and BTZ was evaluated by MTT assays in MM-CSCs and by XTT assays in RPMIs, MM1.S, and MM1.R cells as described in “Materials and Methods.” Results are the mean of three independent experiments ± SEM. ^b^Positive controls: WFA and BTZ. ^∗^Significantly higher than the corresponding values in control sensitive cells as determined by an unpaired *t*-test (*p* < 0.05).*

### WND Induced Cell Death and Apoptosis in Drug-Sensitive and Drug-Resistant MM Cells

**FIGURE 2:**
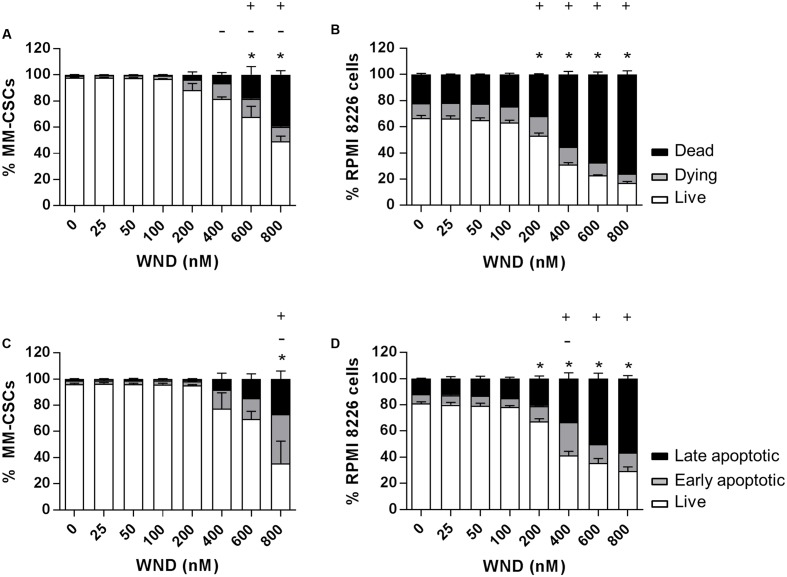
Effects of WND on MM cell viability and apoptosis. Cells were treated with vehicle control or increasing concentrations of WND for 24 h, and then stained with calcein/EH for cell viability **(A,B)**, or AV/PI for apoptosis **(C,D)** as described in “Materials and Methods.” Results represent the means ± SEM of at least three independent experiments. Percentage of live (^∗^), dying (–), and dead (+) cells; or live (^∗^), early apoptotic (–), and late apoptotic (+) cells significantly different from control values as determined by a one-way ANOVA, followed by Bonferroni multiple comparison test (*p* < 0.05).

The cytotoxic effect of a 24 h treatment with WND was measured by flow cytometric analyses of calcein/EH staining in MM-CSCs and RPMIs (**Figures 2A,B**). The percentage of viable (calcein^+^/EH^-^) MM-CSCs was significantly reduced from 97.7% with vehicle control treatment to 67.9 and 49.4% with 600 and 800 nM WND, respectively. The percentage of dying (calcein^+^/EH^+^) MM-CSCs significantly increased starting at 400 nM WND, and that of dead (calcein^-^/EH^+^) MM-CSCs significantly increased starting at 600 nM WND. Similarly, RPMIs viability was reduced from 80.9% with vehicle control to 67.2, 41.2, 35.5, and 29.4% with 200, 400, 600, and 800 nM WND, respectively. The percentage of dying RPMIs did not appear to significantly change with increasing doses of WND; however, the percentage of dead RPMIs significantly increased starting at 200 nM.

The effect of WND on CD34^+^ hematopoietic NSCs viability was assessed to determine if WND exhibited any degree of selectivity toward MM-CSCs. The IC_50_ value of WND with respect to viability (calcein^+^ cells) was calculated in MM-CSCs and NSCs after a 72 h treatment with WND. The IC_50_ value was 0.28 ± 0.06 μM in MM-CSCs and greater than 0.8 μM in NSCs, suggesting a minimum of 2.75-fold selectivity. The cytotoxic effect on MM-CSCs and RPMIs prompted us to investigate whether the mechanism of cell death in response to WND was due to apoptosis. Flow cytometric analyses of AV/PI staining showed significant apoptotic effects in both MM-CSCs and RPMIs in response to a 24 h WND treatment (**Figures 2C,D**). The percentage of viable cells (AV^+^/PI^-^) was significantly reduced with 800 nM WND in MM-CSCs, and starting with 200 nM WND in RPMIs. The percentage of apoptotic cells (AV^+^/PI^+^ and AV^-^/PI^+^) was significantly increased with 800 nM WND in MM-CSCs, and starting with 400 nM WND in RPMIs.

### P-gp Inhibition Has No Effect on WND-Induced MM Cell Growth Control

**FIGURE 3:**
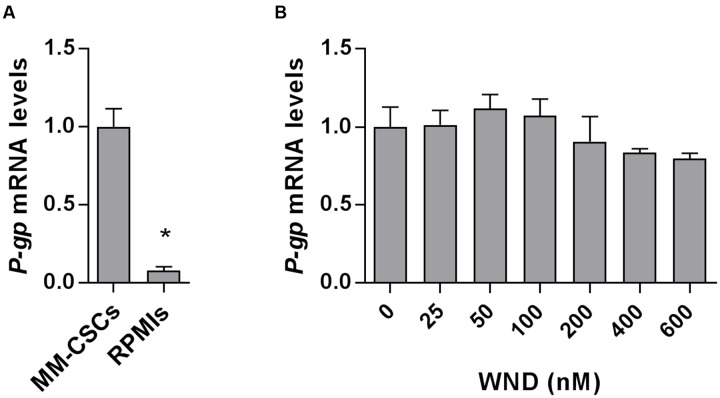
Messenger RNA level of P-gp in MM-CSCs and RPMIs. **(A)** Relative mRNA expression of *P-gp* in MM-CSCs and RPMIs. **(B)** *P-gp* mRNA expression in MM-CSCs following treatment with WND for 24 h. Each bar represents the mean ± SEM of three independent experiments. ^∗^Significantly different from MM-CSCs **(A)**, or vehicle control **(B)** as determined by *t*-test **(A)** or by one-way ANOVA **(B)** followed by Bonferroni multiple comparison test (*p* < 0.05).

Next, the mRNA expression of *P-gp* in MM-CSCs and in RPMIs was compared (**Figure 3A**). Results showed that *P-gp* expression was 12.5-fold lower in RPMIs than in MM-CSCs. In addition, a 24 h WND treatment in MM-CSCs did not affect *P-gp* mRNA expression at the tested doses (**Figure 3B**). The observation that MM-CSCs are resistant to the cytostatic effects of BTZ and WFA, but not to WND, combined with the observation that MM-CSCs highly expresses *P-gp* in comparison to RPMIs, led to investigate whether the antiproliferative activity of BTZ, WFA, and WND was dependent on the function of P-gp efflux transporter. The ability of VRP, a known P-gp inhibitor, to sensitize MM-CSCs to BTZ, WFA, and WND was then measured. A 5 μM VRP treatment sensitized MM-CSCs to both BTZ and WFA, but not to WND (**Table 2**). This was evidenced by the significant reduction in IC_50_ values with both WFA and BTZ after VRP treatment. In contrast, the IC_50_ value of WND was similar with or without VRP (**Table 2**). In RPMIs, which express substantially lower P-gp mRNA levels, the use of VRP did not significantly lower the IC_50_ values (**Table 2**).

**Table 2:** Antiproliferative activity (IC_50_ in nM) of WND, WFA, and BTZ in MM-CSCs and RPMIs with or without VRP.^a^

Compound	MM-CSCs		RPMIs	
		
	DMSO	5 μM VRP	DMSO	5 μM VRP
WND	67.4 ± 12.8	53.7 ± 16.9	69.5 ± 6.7	59.3 ± 5.8
WFA^b^	521.0 ± 20.6	307.2 ± 35.1^∗^	275.7 ± 37.1	218.3 ± 23.3
BTZ^b^	7.6 ± 0.7	3.5 ± 0.3^∗^	4.1 ± 0.3	4.4 ± 0.5


*^a^The antiproliferative activity was measured by MTT assays in MM-CSCs and by XTT assays in RPMIs as described in “Materials and Methods.” Results are the mean of three independent experiments ± SEM. ^b^Positive controls: WFA and BTZ. ^∗^Significantly lower than the corresponding vehicle control treatment as determined by an unpaired *t*-test (*p* < 0.05).*

### WND Did Not Inhibit the Efflux of P-gp Transporter Substrate R123 at Moderate Doses

**FIGURE 4:**
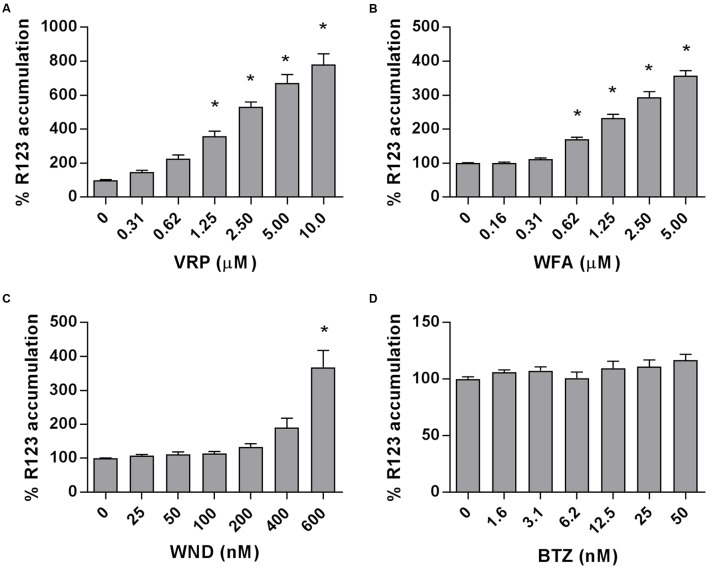
Rhodamine 123 accumulation in MM-CSCs in response to withanolides and controls. R123 accumulation experiments in MM-CSCs were conducted as described in “Materials and Methods.” MM-CSCs were co-treated with R123 and increasing concentrations of VRP **(A)**, WFA **(B)**, WND **(C)**, and BTZ **(D)** for 2 h, followed by the quantification of intracellular R123. Each bar represents the mean ± SEM of three independent experiments. ^∗^Significantly different from vehicle control values as determined by a one-way ANOVA, followed by Bonferroni multiple comparison test (*p* < 0.05).

The intracellular accumulation of R123, a substrate of P-gp transporter, was measured in the P-gp-highly expressing MM-CSCs and was found to significantly increase in a dose-dependent manner in MM-CSCs treated with VRP, starting at a dose of 1.25 μM (**Figure 4A**). Similarly, WFA caused a significant dose-dependent increase in R123 accumulation starting at 0.62 μM (**Figure 4B**). WND did not appear to affect the R123 intracellular accumulation up until 400 nM. However, a 600 nM WND treatment significantly increased R123 intracellular accumulation (**Figure 4C**). BTZ, a molecule previously shown to be affected by P-gp-mediated resistance (O’Connor et al., 2013), did not affect R123 accumulation at the tested doses (**Figure 4D**).

## Discussion

Multiple myeloma patients diagnosed with relapsed/refractory MM face an unfavorable prognosis represented by an overall survival limited to 9 months (Abraham et al., 2015). Recent advances in R/R-MM treatment include the introduction of the immunomodulator pomalidomide and the proteasome inhibitor carfilzomib. The inevitable treatment failure is attributed to the inefficacy of novel therapeutic options to eradicate MM-CSCs (Gao et al., 2016). MM-CSCs are characterized with an efficient efflux capability, rendering them highly resistant to virtually all available treatment options (Gao et al., 2016). MM-CSCs have been shown to highly express P-gp, multidrug resistance protein 1, and breast cancer resistance protein (Gao et al., 2016). In addition, MM-CSCs not only express ABC efflux transporters but also prevail and increase in frequency after chemotherapy (Gao et al., 2016).

Our previous results demonstrated that WFA potently inhibited the growth, as well as induced cell death and differentiation in MM-CSCs. However, the IC_50_ value for the growth inhibitory effect in the drug-resistant MM-CSCs was significantly higher than that in the drug-sensitive tumoral PCs. Given the increasing incidence of cancer chemotherapy resistance, and the frequent relapses associated with multidrug resistance (Lee and Choi, 2016), a search for withanolide analogs that could similarly inhibit the growth of both drug-sensitive PCs and drug-resistant MM-CSCs was initiated. This led to the identification of WND, which exhibited similar growth inhibitory effects in both sensitive and resistant myeloma cells, as shown in two models, the CSCs model and the DEX-resistant model (**Table 1**). Furthermore, WND not only exhibited cytostatic effects, but also cytotoxic activity. This was evidenced by the observation that WND inflicted cell death in both MM-CSCs and RPMIs in assays involving flow cytometric analysis using the viability staining calcein and the mortality staining EH (**Figure 2**).

One important aspect for targeting CSCs is the identification of compounds that selectively affect CSCs while leaving NSCs relatively unaffected. NSCs are crucial for the normal function of the body including hematopoiesis, wound healing, and repair (Shackleton, 2010; Munoz et al., 2012; Nguyen et al., 2012; Medema, 2013). Although WFA only showed a limited degree of selectivity toward MM-CSCs (Issa and Cuendet, 2017), it was valuable to examine to which extent WND exerted selectivity. High WND doses significantly reduced the viability of MM-CSCs, while NSCs appeared resistant to the cytotoxic effects of WND and remained viable at the tested doses. In addition, previously published results showed that WFA did not cause a dose-dependent inhibition of MM-CSCs migration (Issa and Cuendet, 2017). This behavior was also observed with WND (data not shown). However, with respect to differentiation, WFA induced differentiation in MM-CSCs, while this did not seem to be the case with WND. The WFA differentiation effect was accompanied by significant increases in the mRNA expression of *CD44*, *Kit*, *Notch1*, and *HES1* (Issa and Cuendet, 2017). WND did not cause any significant alteration in the mRNA expression of *CD44*, *Kit* or *HES1*, and only minor alterations in *Notch1* (data not shown). Taken together, these results indicate that WND and WFA may exhibit their cytotoxic activity through different mechanisms of action.

Although previous studies showed that WFA could target ovarian and breast CSCs (Suman et al., 2013), these studies did not address the extent of resistance that CSCs exhibited relative to the tumoral cells. RPMIs are non-tumorigenic PCs previously demonstrated to contain a 2–5% subpopulation of tumorigenic cells (Matsui et al., 2004). The present study used a myeloma CSCs model derived from the bone marrow of a MM patient. MM-CSCs have been previously shown to be resistant toward conventional myeloma therapies including the proteasome inhibitor BTZ and the immunomodulatory agent DEX (Issa and Cuendet, 2017). This may be due to MM-CSCs being equipped with detoxification tools rendering them highly resistant to conventional therapies (Huff and Matsui, 2008; Singh and Settleman, 2010; Gao et al., 2016). Several mechanisms of resistance have already been identified in MM-CSCs, and these include increased DNA repair, decreased activation of the apoptotic machinery, increased activity of the detoxification enzyme known as aldehyde dehydrogenase, and increase in the efflux of xenobiotics (Huff and Matsui, 2008; Gao et al., 2016).

Since ABC efflux transporters offer protection against xenobiotics, one interesting strategy proposed for the eradication of resistant cancer cells was the inhibition of ABC efflux transporters. Several generations of efflux transporter inhibitors have been developed, but due to severe adverse effects these inhibitors were rendered clinically unsuitable (Dean et al., 2005; Falasca and Linton, 2012). However, an alternative strategy to the inhibition of ABC efflux transporters is the identification of molecules with an anticancer activity independent of the function of ABC efflux transporters. Therefore, compounds with a cytostatic effect similar between MM-CSCs and tumoral PCs would be desirable. The results of the present study showed that WND not only inhibited the growth of both drug-sensitive PCs and drug-resistant MM-CSCs (which highly express P-gp, **Figure 3A**), but also the use of the P-gp inhibitor, VRP, did not modify the cytostatic effect of WND in MM-CSCs (**Table 2**). This indicates that the growth inhibitory effect of WND in MM-CSCs may be independent of the efflux activity of P-gp.

Multiple myeloma treatment currently utilizes combination therapies (Rajkumar, 2011). One unfavorable aspect of combination therapy is the susceptibility to drug–drug interactions and potentially adverse effects (Gottesman, 2002). Since the inhibition of ABC efflux transporter may result in serious adverse effects, it was important to evaluate if WND inhibited P-gp. The results showed that WND did not increase the concentration of the P-gp substrate, R123, in MM-CSCs at moderate WND doses (0–400 nM) indicating that WND may not act as a P-gp inhibitor at doses relevant for its cytostatic activity (**Figure 4**). This is a desired effect if WND is used in combination therapy as it should be less susceptible to drug–drug interactions observed with classical P-gp inhibitors. In addition, WND did not affect the mRNA expression of *P-gp* suggesting that the lack of effect on the efflux activity of P-gp is not due to a reduction in gene expression at the tested doses (**Figure 3B**). This further supports that WND may not interfere with the efflux of other P-gp-dependent conventional therapies such as BTZ due to a reduction of *P-gp* expression (O’Connor et al., 2013).

The identification of compounds capable of eradicating resistant MM-CSCs is urgently needed as it may improve the prognosis of MM patients (Huff and Matsui, 2008; Agarwal and Matsui, 2010; Gao et al., 2016). In the present study, WND is proposed as a lead molecule with similar cytostatic activity in both sensitive and resistant MM cells. In addition, the efflux activity of P-gp did not affect the cytostatic activity of WND in both sensitive and resistant MM cells, and WND did not interfere with the function of P-gp. Such molecules are urgently required and therefore, the results obtained in this study warrant further investigation of WND and its analogs in relevant MM animal models.

## Author Contributions

MI, AG, and MC designed the research. MI and EW conducted the experiments and analyzed the data. MI, AG, and MC wrote the manuscript. All authors read and approved the final manuscript.

## Conflict of Interest Statement

The authors declare that the research was conducted in the absence of any commercial or financial relationships that could be construed as a potential conflict of interest.

## Abbreviations

ANOVA: analysis of variance
AV: annexin V
BTZ: bortezomib
calcein: calcein AM
CSCs: cancer stem cells
DEX: dexamethasone
EH: ethidium homodimer
FBS: fetal bovine serum
MM: multiple myeloma
NMR: nuclear magnetic resonance
NSCs: normal stem cells
PCs: plasma cells
P-gp: P-glycoprotein
PI: propidium iodide
R/R-MM: relapsed and refractory multiple myeloma
RPMIs: RPMI 8226 cells
R123: rhodamine 123
SEM: standard error of the mean
TLC: thin-layer chromatography
VRP: verapamil
WFA: withaferin A
WND: withanolide D
